# The complete chloroplast genomes of two species of *Zygophyllum* (Zygophyllaceae)

**DOI:** 10.1080/23802359.2020.1825132

**Published:** 2020-10-05

**Authors:** Haoming Xu, Weirui Fu, Wei Xie, Yuguo Wang, Yunfei Zhang

**Affiliations:** a Ministry of Education Key Laboratory for Biodiversity Science and Ecological Engineering, Institute of Biodiversity Science, School of Life Sciences, Fudan University, Shanghai, China; b Natural History Research Centre of Shanghai Natural History Museum, Shanghai Science & Technology Museum, Shanghai, China

**Keywords:** *Zygophyllum*, chloroplast genome, phylogenetic analysis

## Abstract

*Zygophyllum xanthoxylon* and *Z. fabago* are two important desert plants from Zygophyllaceae, which are both widely distributed in north-western China. Here, we report the complete chloroplast genome sequences of *Z. xanthoxylon* and *Z. fabago*, which are 109,577 bp and 108,695 bp in length, respectively. The inverted repeat regions, the large single-copy region and the small single-copy region of *Z. xanthoxylon* are 5084 bp, 83,735 bp, and 15,674 bp in length, respectively, while those of *Z. fabago* are 4669 bp, 82,293 bp and 17,064 bp in length, respectively. A total of 98 genes were annotated in the genome of *Z. xanthoxylon* including 29 tRNA, 4 rRNA and 65 protein-coding genes, and 100 genes were annotated in the genome of *Z. fabago* including 31 tRNA, 4 rRNA and 65 protein-coding genes. Phylogenetic analysis showed *Z. xanthoxylon* clustered and *Z. fabago* formed a monophyletic group sister to *Tetraena mongolica*.

The genus *Zygophyllum* are widely distributed in the arid and semi-arid areas of Africa, Europe, Asia and Australia. In the desert of north-western China, the shrub *Zygophyllum xanthoxylon* and the herb *Z. fabago* have ever been proposed as members of two distinct genera, *Sarcozygium* and *Zygophyllum*, in Zygophyllaceae (Liu 1998). However, phylogenetic evidence from chloroplast gene *rbcL* and *trnL-F* (Han et al. 2016) supported that *Sarcozygium* was merged into *Zygophyllum* (Liu and Zhou 2008). The Zygophyllaceae species including *Zygophyllum* and *Tetraena* are important constituents of local plant community (Wan et al. 2006; Ma et al. 2019), with key ecological functions on sand fixation and preventing desertification. To better understand the phylogenetic relationship and evolution of the species in Zygophyllaceae, we sequenced the complete chloroplast genome of *Z. xanthoxylon* and *Z. fabago* and compared them with the related species.

The DNA of *Z. xanthoxylon* and *Z. fabago* was extracted from the fresh leaves which were collected on the north-western Ordos plateau in Inner Mongolia, China. The voucher specimens were deposited in the herbarium of Fudan University (FUS). Fresh leaves dried with silica-gel were used to extract total genomic DNA, which sequenced using the Illumina Hiseq 2500 (Illumina, San Diego, CA, USA), with 150 bp paired-end sequencing. The two complete chloroplast genomes were assembled by SOAPdenovo2 v2.04 (Luo et al. 2012) and Bowtie2 v2.3.4.1 (Langmead and Salzberg 2012). GapCloser v2.04 (Luo et al. 2012) was used to fill the gaps; MUMmer v3.23 (Kurtz et al. 2004) was used for linear alignment; and DOGMA was used for annotation (Wyman et al. 2004). The two complete chloroplast genome sequences have been submitted to NCBI (https://www.ncbi.nlm.nih.gov, GenBank accession numbers: MT796491 and MT796492). Using RAxML (Stamatakis 2014), a maximum likelihood (ML) analysis was conducted for eight species from Zygophyllales including *Z. xanthoxylon* and *Z. fabago,* and 20 representative species from nine orders.

The total length of the complete chloroplast genome of *Z. xanthoxylon* is 109,577 bp and that of *Z. fabago* is 108,695 bp. In *Z. xanthoxylon*, the typical quadripartite structure includes an LSC region of 83,735 bp, an SSC region of 15,674 bp and a pair of IRs of 5084 bp. In *Z. fabago*, the length of corresponding regions are 82,293 bp, 17,064 bp and 4669 bp, respectively. The GC content of two chloroplast genomes are 33.77% and 33.73%. A total of 98 predicted genes including 65 protein-coding genes, 29 tRNA genes and 4 rRNA genes were identified in *Z. xanthoxylon* and a total of 100 predicted genes including 65 protein-coding genes, 31 tRNA genes and 4 rRNA genes were identified in *Z. fabago*.

The phylogenetic analysis showed that *Z. xanthoxylon* and *Z. fabago* formed a monophyletic group which cluster with *T. mongolica* (Figure 1). Besides, our result confirmed that Zygophyllaceae had a closer relationship with other Fabidae taxa than with Nitrariaceae in Sapindales (Dong et al. 2019).

**Figure 1.**
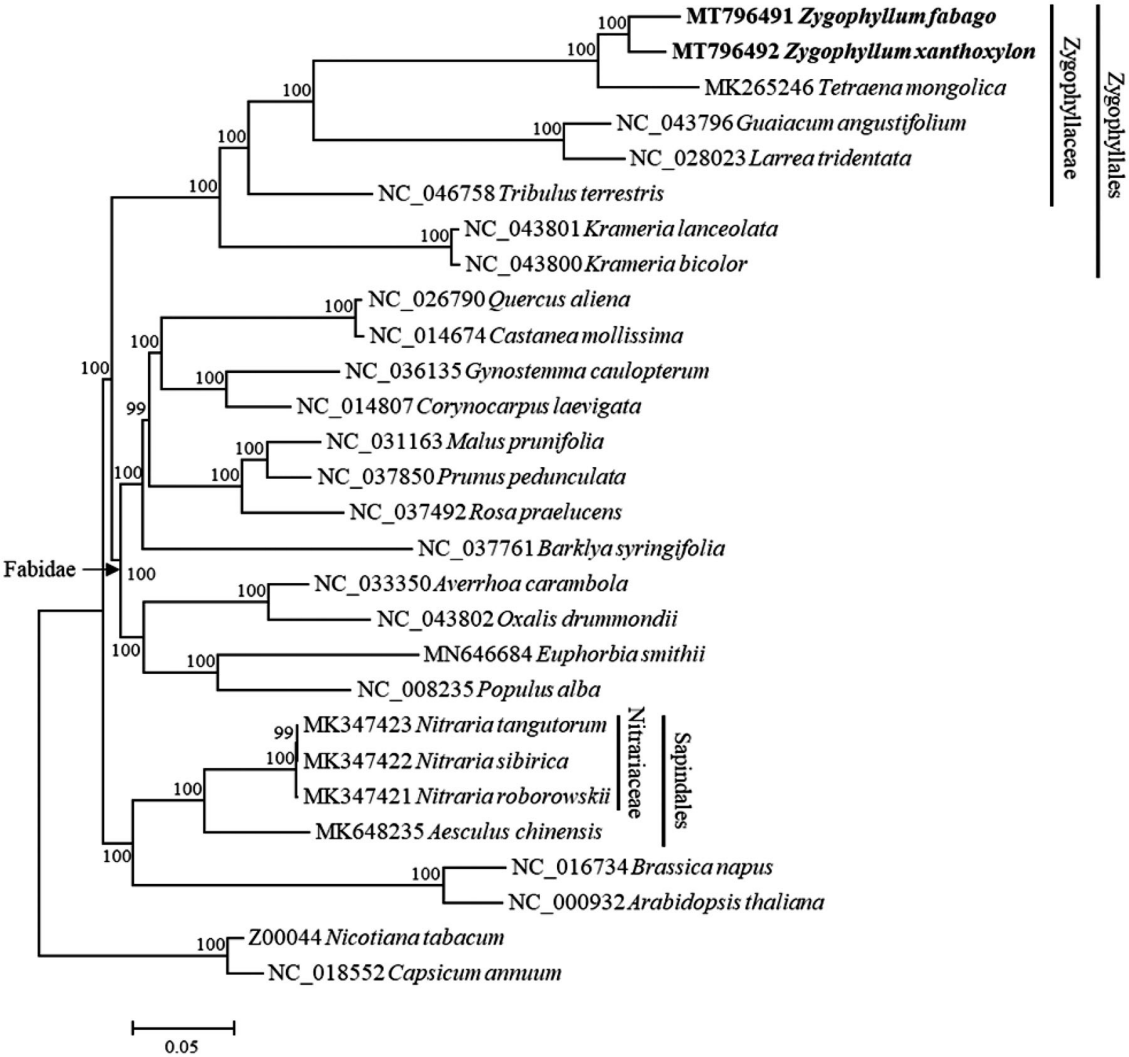
Maximum likelihood phylogenetic tree based on the chloroplast genome of 28 species. Numbers above the branches indicate bootstrap support values (1000 replicates).

## Data Availability

The data that support the findings of this study are openly available in NCBI GenBank at https://www.ncbi.nlm.nih.gov, reference number MT796491 and MT796492. Illumina raw sequencing data for two *Zygophyllum* species have been deposited in the NCBI Sequence Read Archive (SRA) under accession SRR12506738 and SRR12506739.
